# “Take up to eight tablets per day”: Incorporating free‐text medication instructions into a transparent and reproducible process for preparing drug exposure data for pharmacoepidemiology

**DOI:** 10.1002/pds.5595

**Published:** 2023-02-11

**Authors:** Meghna Jani, Belay Birlie Yimer, David Selby, Mark Lunt, Goran Nenadic, William G. Dixon

**Affiliations:** ^1^ Centre for Epidemiology Versus Arthritis Centre for Musculoskeletal Research, The University of Manchester Manchester UK; ^2^ NIHR Manchester Biomedical Research Centre Manchester University NHS Foundation Trust, Manchester Academic Health Science Centre Manchester UK; ^3^ Salford Royal Hospital Northern Care Alliance NHS Foundation Trust Salford UK; ^4^ Department of Computer Science University of Manchester Manchester UK

**Keywords:** drug exposure definition, electronic health records, free text, misclassification, narrative prescribing instructions, opioids, pharmacoepidemiology

## Abstract

**Purpose:**

Routinely collected prescription data provides drug exposure information for pharmacoepidemiology, informing start/stop dates and dosage. Prescribing information includes structured data and unstructured free‐text instructions, which can include inherent variability, such as “one to two tablets up to four times a day”. Preparing drug exposure data from raw prescriptions to a research ready dataset is rarely fully reported, yet assumptions have considerable implications for pharmacoepidemiology. This may have bigger consequences for “pro re nata” (PRN) drugs. Our aim was, using a worked example of opioids and fracture risk, to examine the impact of incorporating narrative prescribing instructions and subsequent drug preparation assumptions on adverse event rates.

**Methods:**

R‐packages for extracting free‐text medication prescription instructions in a structured form (*doseminer*) and an algorithm for transparently processing drug exposure information (*drugprepr*) were developed. Clinical Practice Research Datalink GOLD was used to define a cohort of adult new opioid users without prior cancer. A retrospective cohort study was performed using data between January 1, 2017 and July 31, 2018. We tested the impact of varying drug preparation assumptions by estimating the risk of opioids on fracture risk using Cox proportional hazards models.

**Results:**

During the study window, 60 394 patients were identified with 190 754 opioid prescriptions. Free‐text prescribing instruction variability, where there was flexibility in the number of tablets to be administered, was present in 42% prescriptions. Variations in the decisions made during preparing raw data for analysis led to marked differences impacting the event number (*n* = 303–415) and person years of drug exposure (5619–9832). The distribution of hazard ratios as a function of the decisions ranged from 2.71 (95% CI: 2.31, 3.18) to 3.24 (2.76, 3.82).

**Conclusions:**

Assumptions made during the drug preparation process, especially for those with variability in prescription instructions, can impact results of subsequent risk estimates. The developed R packages can improve transparency related to drug preparation assumptions, in line with best practice advocated by international pharmacoepidemiology guidelines.

**Key Points**

- Open‐source software in R to extract numeric dosage information from free‐text prescribing instructions (*doseminer*) and prepare prescribing drug data to derive drug exposure transparently (*drugprepr*) was developed.
- The impact of varying drug preparation assumptions using the example of opioid use on fracture risk was tested.
- In 60 394 patients with 190 754 opioid prescriptions, 42% of prescriptions had some variability within instructions.
- Changing drug preparation assumptions impacted events (*n* = 303–415), person years of exposure (5619–9832) and the distribution of risk associated with the drug: hazard ratio 2.71 (95% CI: 2.31, 3.18) to 3.24 (2.76, 3.82).
- By sharing the newly developed software source code, this can be further developed by the pharmacoepidemiology community to be used in a wider range of settings and to incorporate further complexities in drug exposure data preparation.

**Plain English Summary:**

Electronic prescriptions contain key information to allow calculation of how long a patient is exposed to a drug, needed for drug safety analyses. Prescription instructions often contain variability, for exaxmple, “Take one to two tablets up to four times a day”, as needed by the patient. How researchers prepare this information for drug safety research can have implications on the final results, however, are rarely reported. Challenges include such instructions are “unstructured” or not machine readable for analysis and lack of clear processes on how to prepare such data reproducibly. Our aim was to examine the impact of incorporating information from such instructions and subsequent drug preparation assumptions in drug safety research, using the case study of opioid use on fracture risk. We developed software packages for extracting text information within such instructions and converting them to numbers (*doseminer*), as well as transparently processing prescriptions before performing analysis (*drugprepr*). We used national general practice records to test how varying drug preparation assumptions influences the risk of opioid use on fracture risk. Our results show that 42% of opioid prescriptions had instruction variability. Variation in the decisions led to marked differences affecting the numbers of fractures (*n* = 303–415) on drug, time on medication (5619–9832 person years), and eventual fracture risk associated with opioids. The open‐source software packages developed have the potential to improve the efficiency, transparency, and reproducibility of drug safety research in line with international recommendations.

## 1 BACKGROUND

Routinely collected health data from electronic health records (EHRs) are an important source of real‐world evidence to study the effectiveness and safety of medications.^1^ Real‐world evidence has great potential to inform clinical guidance^2^, ^3^ though requires robust methodology and clear definitions of all exposures, confounders and outcomes to remain trustworthy and reproducible.^4^ Information on drug exposure is usually derived from electronic prescribing records, which include enough detail to guide dispensing to the patient. For research purposes, however, it may not always include precise information such as the prescription stop dates or daily dose required to derive exposure periods.^5^ These need to be derived based on a series of assumptions, leading to potential exposure misclassification and a lack of reproducibility if not reported.^6^, ^7^ The prescription instructions may also include flexibility for how medications are taken (described in more detail in the following sections).

The REporting of studies Conducted using Observational Routinely collected Data for Pharmacoepidemiology (RECORD‐PE) guideline advocates transparency in how drug exposure data is prepared, converting (serial) prescriptions to data ready for analysis.^4^ However, the steps in defining exposure information are rarely fully reported. Assumptions made during this stage have considerable implications for risk attribution of possible adverse events (AEs).^8^ We have previously developed an algorithm for improving the transparency and reporting of drug data preparation (called ‘drug prep’ using STATA®), and explored the impact of assumptions made in data preparation using coded structured prescription data.^8^ The algorithm includes a series of 10 decision nodes with 54 possible assumptions that can be pre‐specified by the researcher during drug data preparation. For instance, the algorithm allows the researcher to re‐set missing or implausible values to individual or population level means, use previous values from that individual, or other transparent assumptions as appropriate. In an applied example of assessing cardiovascular events in diabetic patients following exposure to oral sulphonylureas compared to biguanides, we found that the decisions made during the drug preparation stage led to wide variability in hazard ratios (from 1.77 [95% CI: 1.56–2.00] to 2.83 [95% CI: 1.59–5.04.]).^8^ Whilst this example demonstrated that decisions made during drug data preparation could influence consequent results, it did not deal with free‐text prescription instructions, which is a particularly important issue for “as required” prescriptions.

Narrative electronic prescribing instructions are free‐text excerpts that convey additional information on the administration of a medication as directed by the prescriber. They are often not used in research, however, due to their unstructured nature, which makes them less machine readable and thus unsuitable for direct use in modelling. Still, they contain valuable material that have the potential to advance our understanding of drug safety. Often prescription data instructions can offer a choice of dose frequency to the patient. For instance, “Take one to two tablets up to four times a day”, offers a patient choice of taking between one and eight tablets per day. Such “pro re nata” or PRN drugs are used especially for treating pain, nausea, indigestion, anxiety, insomnia, or other chronic conditions with time‐varying symptoms. Patients may reasonably administer medications at either end of these ranges depending on the extent of their symptoms. Pharmacoepidemiology studies tend to have followed opaque assumptions, either in the researchers' own data preparation or prior to receiving the data from action by the research database custodian.^9^, ^10^ The ensuing impact can lead to failure to reproduce results using the same data‐source, and more broadly reduce trust in real‐world evidence potentially diminishing their influence to drive improvements in population health.

We have performed prior work in text‐mining dosage instructions to allow pharmacoepidemiology studies to handle free‐text instructions, outputting a machine‐readable range of possible exposures for any single prescription.^11^ This allows researcher‐led interpretation of possible ranges of exposure derived from instructions such as “one to two tablets up to four times a day.” However, there are challenges in applying such algorithms due to different datasets, different analytical software, regulatory issues and lack of standardization of narrative electronic prescribing instructions.^12^ We have recently updated our narrative prescribing text‐mining algorithm as an R package “Doseminer” and also released an R package version of our prior STATA “DrugPrep” algorithm, with accompanying example datasets and short vignettes (*doseminer* and *drugprepr* are both freely available on CRAN). Together, these two linked R packages have the potential to improve the efficiency and transparency of drug exposure data preparation, including the incorporation of free‐text prescribing instructions. In order to motivate its use, it is necessary to examine and illustrate the possible implications of decisions made in the drug data preparation process, especially for medications with inherent uncertainty in the dosage instructions.

The aims of this work therefore were to (1) introduce R packages for drug data preparation, including the processing of free‐text dosage instructions to derive more accurate exposure history; and (2) examine the implications of incorporating narrative prescribing instructions and subsequent drug data preparation assumptions on adverse event rates, using the worked example of opioids and fracture risk using data from primary care records via the Clinical Practice Research Datalink (CPRD). This is intended to show the importance of using free‐text instructions in pharmacoepidemiology and help other researchers incorporate this into their analyses.

## 2 METHODS

### 2.1 Derivation of drug exposure periods

For transparent drug preparation, first the variability within free text instructions of a prescription needs to be considered, prior to passing this information through the drug preparation algorithm. To this end, we developed an *R‐package* (“*doseminer*”)^13^ that extracts and represents free‐text medication prescription instruction information in a structured form following a previously published algorithm,^11^ but which avoids making its own assumptions (e.g., that “one to two tablets per day” equates to 1.5 tablets per day).The R‐package—*doseminer* ^13^ can be installed from CRAN using.

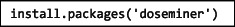

or get the latest development version via GitHub:

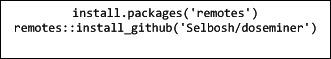

**FIGURE 1:**
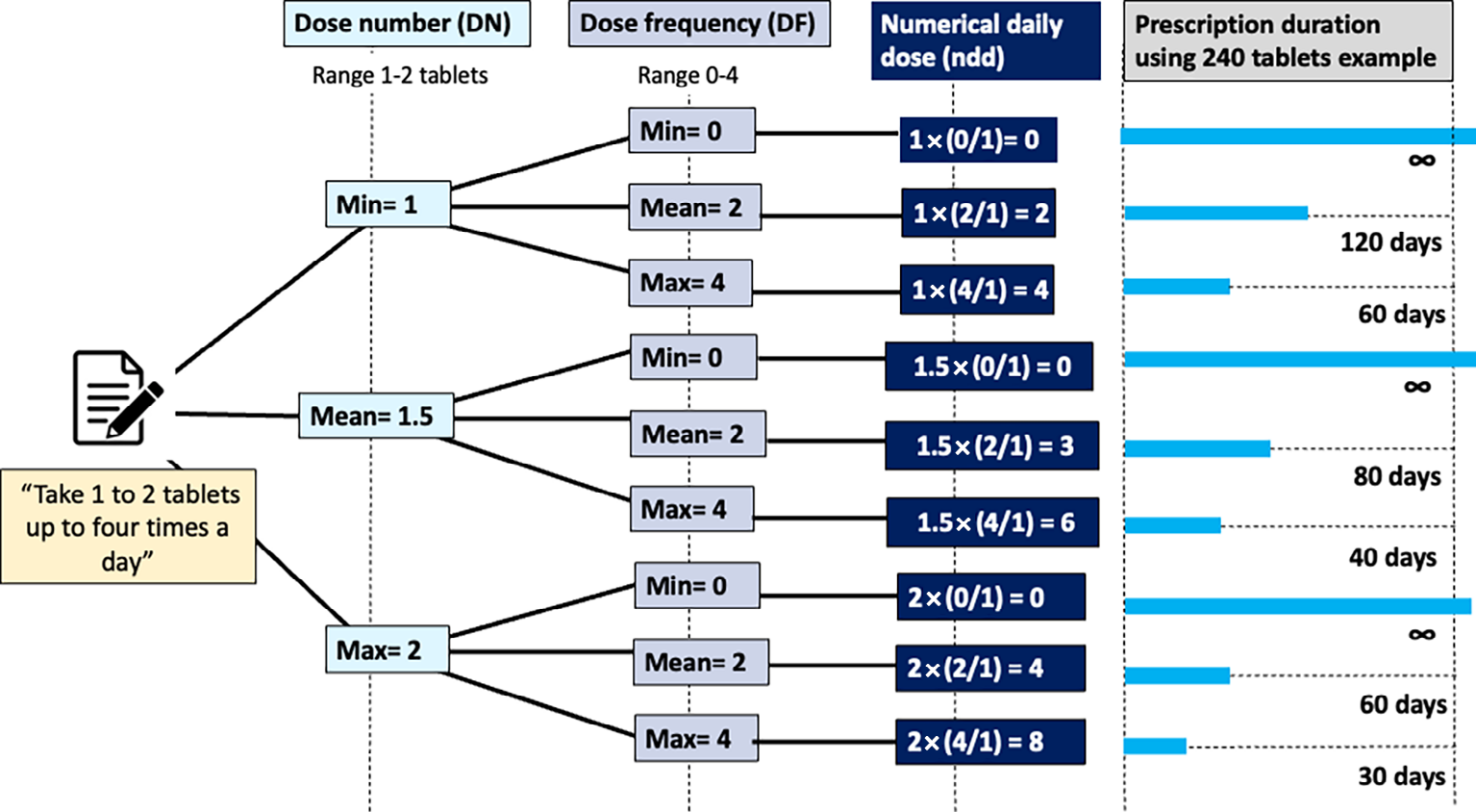
Derived exposure durations for one single prescription. Tree diagram showing sequential choices of DN then DF for the prescription instructions “take 1 to 2 tablets up to four times a day” for 240 dispensed tablets, and the resultant durations of medication use. Although the choice of DF = minimum (i.e., 0) leads to an implausible value, this gets addressed subsequently within DrugPrepR. The quantity of 240 tablets is used for illustrative purposes. DF, drug frequency; DI, drug interval; DN, drug number; max, maximum; min, minimum; ndd, numeric daily dose.

An introduction on how to use the package is available at Pharmacoepidemiology with *doseminer* (r‐project.org).^14^ A flow diagram of this algorithm demonstrating the steps undertaken to clean and extract the free text prescription is presented in Figure S1. The algorithm represents each free‐text prescription using three key metrics: dose number ([DN], number of medication units taken in a single administration), dose frequency ([DF], the number of times the dose is taken in the dose interval), and dose interval ([DI], the time interval to which the dose frequency applies). There is a need to be careful with the terminology with more detailed definitions in the above package. Moreover, it represents the variability and flexibility in drug directions by including minimum and maximum values for DN, DF and DI of administration, which in turn allow researchers to make transparent choices. For example, the common text instruction “take one to two tablets up to four times a day” is represented as a minimum DN of 1 and a maximum of 2, a minimum DF of 0 and maximum of 4, and a DI of 1 day (minimum = maximum) with “tablet” as the unit (Figure 1). Together with the quantity prescribed, the extracted numeric prescription dosage attributes can then be used to compute the duration of a given prescription, after making decisions about which value to use within any range for DF, DI, and DN. Figure 1 illustrates how a single prescription with variability in DF and DN can lead to considerable variability in the prescription duration depending on the decisions made. The duration of exposure for a given medication in days is given by the following:

$$\text{Duration}=\frac{\text{Quantity prescribed}\times \mathrm{DI}\;\text{in days}}{\mathrm{DF}\times \mathrm{DN}}=\frac{\text{Quantity prescribed}}{\text{numerical daily dose}\;\left(\mathrm{ndd}\right)}$$

Although this calculation can lead to implausible values when dose frequency are set to zero, this is corrected at subsequent steps (see below). Figure S2 illustrates how prescription duration would vary assuming a minimum drug frequency of 1 rather than 0.

To implement the above exposure duration computation, we developed an open‐source R‐package, *drugprepr*,^15^ available in the CRAN repository and can be installed using.

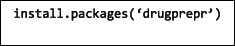

or get the latest development version via GitHub:

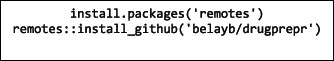

Instructions on how to use the *drugprepr* package are available at Introduction to *drugprepr* (r‐project.org).^16^

An important feature of the *drugprepr* R‐package is its handling of prescriptions where there is variability in drug directions, such as “take one to two tablets up to four tablets per day”. The *drugprer* R‐package provides a function called *compute_ndd*, which allows the researcher to select from the minimum, mean, and/or maximum values of DN, DF, and DI derived from *doseminer* transparently and efficiently to compute ndd and hence the duration of exposure.

**FIGURE 2:**
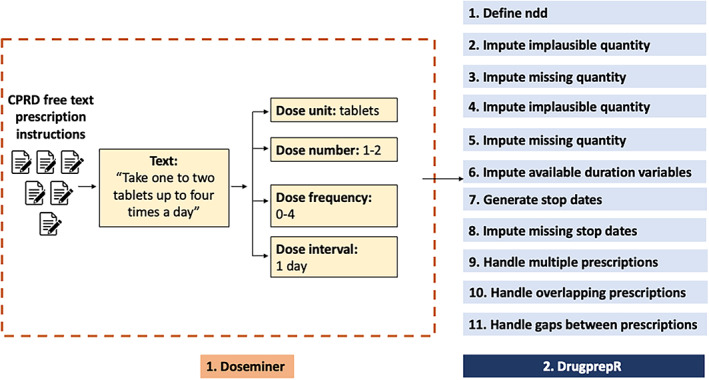
The multi‐step approach for drug preparation from CPRD prescription instructions. *doseminer* extracts numerical values such as dose number and frequency from a free‐text prescription. DrugprepR is the drug preparation algorithm that allows transparent preparation of medication data using the above 11 steps. The data processing options for each steps are listed in Figure 3. CPRD, Clinical Practice Research Datalink; ndd, numeric daily dose

**FIGURE 3:**
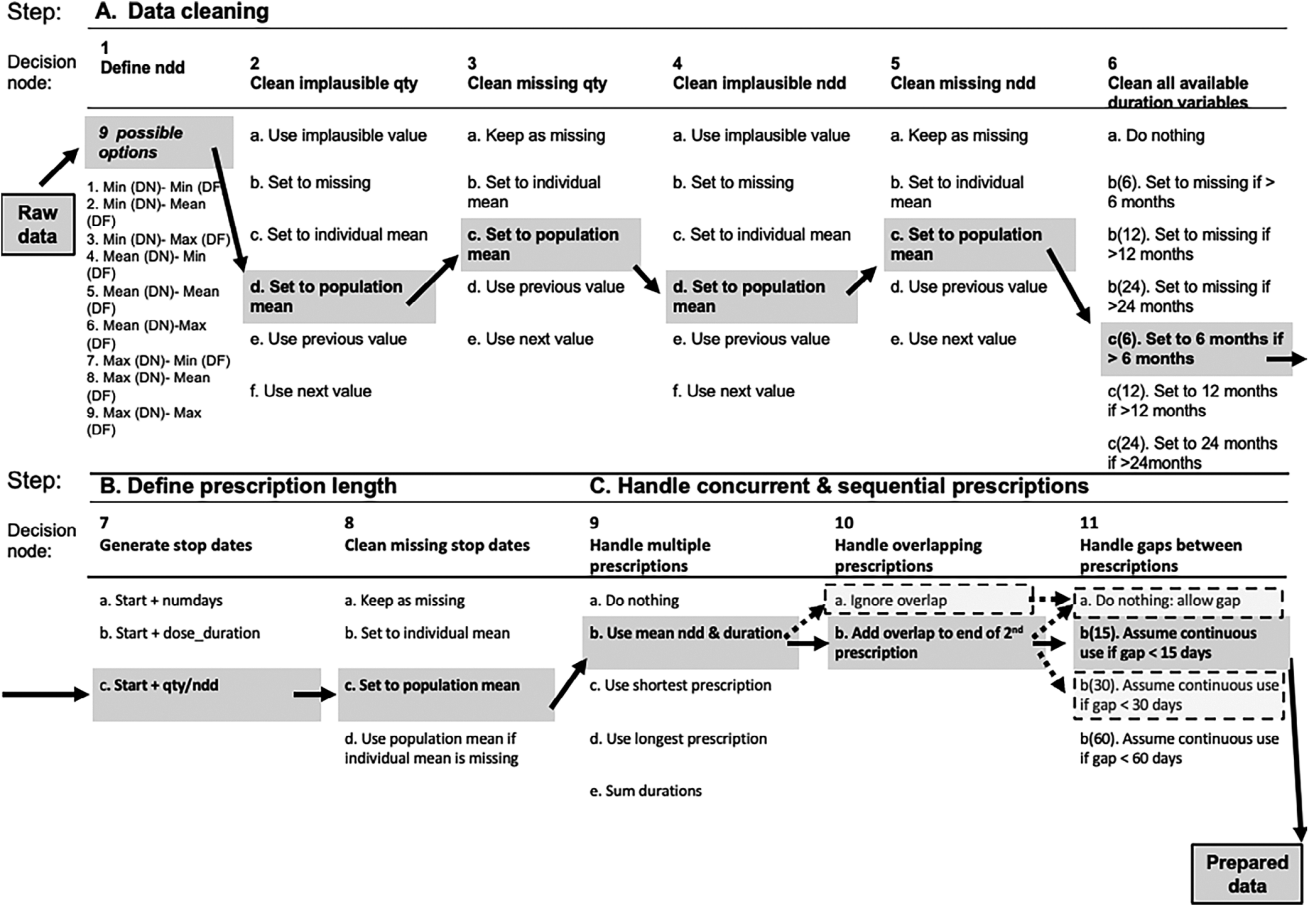
Decisions made to prepare drug exposure data in this study. This figure illustrates the different drug preparation decisions made for the opioids fracture analysis. The options highlighted in the dark gray boxes represent the decisions made for the primary analysis. The options in the dashed light gray boxes represent the decisions altered for the sensitivity analyses performed. For option 10c, while these analyses do not incorporate dose, selecting this option allows this function to be performed if needed for future work. For Decisions 2–5, one can also use median or modal value instead of mean value. The figure has been adapted from Pye et al.^8^ DF, drug frequency; DI, drug interval; DN, drug number; max, maximum; min, minimum; ndd, numeric daily dose.

A typical medication dataset can contain implausible or missing information needed to derive prescription duration. However, this can be adjusted or imputed using other information in the dataset. For example, if every other prescription of a particular formulation in the dataset were for 14 tablets, it would be reasonable to replace a missing quantity with 14. The *drugprepr* package offers a variety of ways to impute missing or replace unreasonable values for the total number of “doses” prescribed (quantity) and the number taken per day (ndd = {DF × DN}/{DI in days}), based on information from other prescriptions from the same individual, the practice the individual belongs to, or the entire sample of prescriptions. It then calculates the start and stop dates for each period of exposure and offers a number of alternatives for handling overlapping prescriptions and ones with brief gaps between them (which the researcher may wish to treat as a continuous period of exposure). Figure 1 illustrates taking a single prescription to the end of step 1 (define ndd) in drug preparation. The subsequent decision steps included in *drugprepr* are presented in Figures 2 and 3. The *drugprepr* package offers the option of selecting individual or population mean, median or mode for decisions 2–5 (Figure 3).

## 3 CASE STUDY

### 3.1 Opioids and fractures

To demonstrate the use of the drug preparation algorithms and the impact of researchers' choices when preparing drug exposure data for drug safety studies, we used CPRD GOLD data to investigate the risk of fracture following opioid prescriptions. CPRD is a well‐established UK database of anonymised primary care EHRs, with information on prescriptions derived from electronic prescribing. The case study of opioids and fracture risk was chosen as opioids are a group of medications commonly prescribed in primary care both in the United Kingdom and internationally.^17^, ^18^, ^19^ Like several analgesics, they can be prescribed “as required” and often have some variability in their prescription instruction. Previous studies that have evaluated the association between opioids and fractures have ignored the assumptions made during the data preparation stage,^9^, ^10^, ^20^, ^21^, ^22^ hence impacting the reproducibility of such findings.

### 3.2 Study setting and population

A retrospective cohort study was performed. The study sample comprised individuals of ≥18 years of age and who had a record of at least one opioid prescription between January 1, 2017, and December 31, 2017. The study sample is a subset of an established cohort of opioid new users without cancer, as described previously.^17^ A 24‐month “wash‐out” period prior to the index date was used to identify new users. Patients with a previous history of a cancer Read code up to 10 years before the index date were excluded, with the exception of non‐melanoma skin cancer.^17^ Individual patients were followed from the date of their first opioid prescription until the date of the first fracture, transfer out of GP practice, GP practice last collection date, death, or July 31, 2018. Codelists and R scripts used to perform this analysis are available via Github.^23^ The study was approved by the CPRD's Independent Scientific Advisory Committee (approval number: 16_278).

### 3.3 Exposure and outcome

Opioid prescriptions were identified through product codes. Oral administration (such as tablets/ pills) and topical routes (e.g., patches) were included as are most commonly prescribed in the United Kingdom. Others such as inhaled or nasal routes we excluded. The data were prepared using the two algorithms described above and the assumptions made are described in the following sections. Person‐time for each individual was classified as “exposed” for the duration of each prescription or “unexposed.” The outcome of interest was a fracture identified using Read codes. The codelist for all fractures included in the analysis is available via Github.^23^ Follow up time was censored at the first event, hence recurrent fractures were not included in this analysis. If fractures occurred on the same date as a prescription start date, these events were excluded from the analysis to avoid reverse causality bias. Events were attributed to opioids if the fracture occurred whilst the patient was actively receiving the drug (an “on drug” analysis). Events occurring when a patient was not on therapy were attributed to unexposed person time. We did not include multiple confounders as the intention was to illustrate the impact of changing data preparation assumptions.

### 3.4 Statistical analysis

Following extraction of numerical values using *doseminer*, we considered nine data preparation pathways associated with the computation of ndd at Step 1 of the *drugprepr* algorithm (Figure 1). We included minimum, maximum and mean values for DN and DF, leading to the nine possible combinations. As the opioid prescriptions used were always prescribed at daily intervals, the minimum, mean, and maximum values of DI was unchanged at a value of one and hence had no variability. As the impact of subsequent decisions on missing/implausible values, overlapping prescriptions, and small gaps between prescriptions has been previously been explored and reported,^8^ we used a fixed decision set for Steps 2–11 for our primary analysis (solid grey boxes in Figure 3). The influence of opioid prescriptions on fracture risk was examined using unadjusted Cox modelling, with subjects off therapy as the referent group. Cox‐modeling was conducted on each dataset, and the HRs and associated confidence intervals were examined graphically. Given that variations in calculating ndd may have led to overlapping prescriptions in patients with multiple prescriptions, we conducted a sensitivity analysis to examine how decisions at this step would influence the results. For this, we limited the cohort to people with multiple opioid prescriptions (>3) and used a range of assumptions at Steps 10 and 11 (dashed light gray boxes in Figure 3) while using a fixed decision set for Steps 2–9 as above. To simplify the presentation, we also fixed DF at its maximum value, DI at the value of one, and considered minimum, maximum and mean values for DN. For Decision 10, we elected to either (a) allow overlap of prescriptions of the same drug (b) shift forward the start date of the prescription to the end date of previous prescription. For Decision 11, we either (a) allowed gaps between prescriptions (i.e., do nothing and consider the participant unexposed for a short period between prescriptions), or (b) assumed continuous use if gaps between prescriptions were less than 30 days.

## 4 RESULTS

**FIGURE 4:**
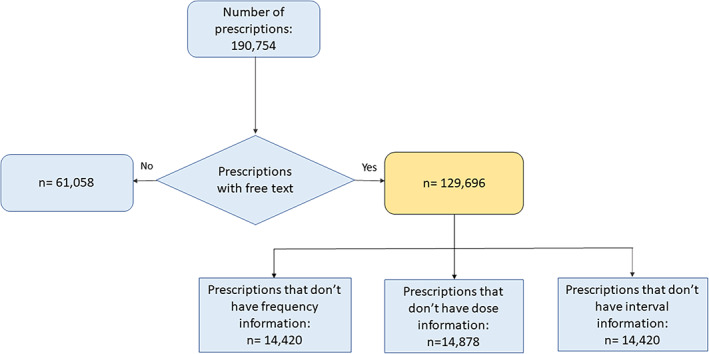
Free text prescription completeness. The absence of free text information meant that the analysis progressed through the usual drug preparation steps as other usual researchers would do without this information.

During the study period a total of 60 394 patients were identified with 190 754 prescriptions for opioid medication. Of the total prescriptions, 41.6% had some variability in the prescribing instructions, introducing scope for alternative (often unreported) assumptions in preparing drug exposure data. Figure 4 shows the proportion of prescription free text completeness in CPRD Gold.

**FIGURE 5:**
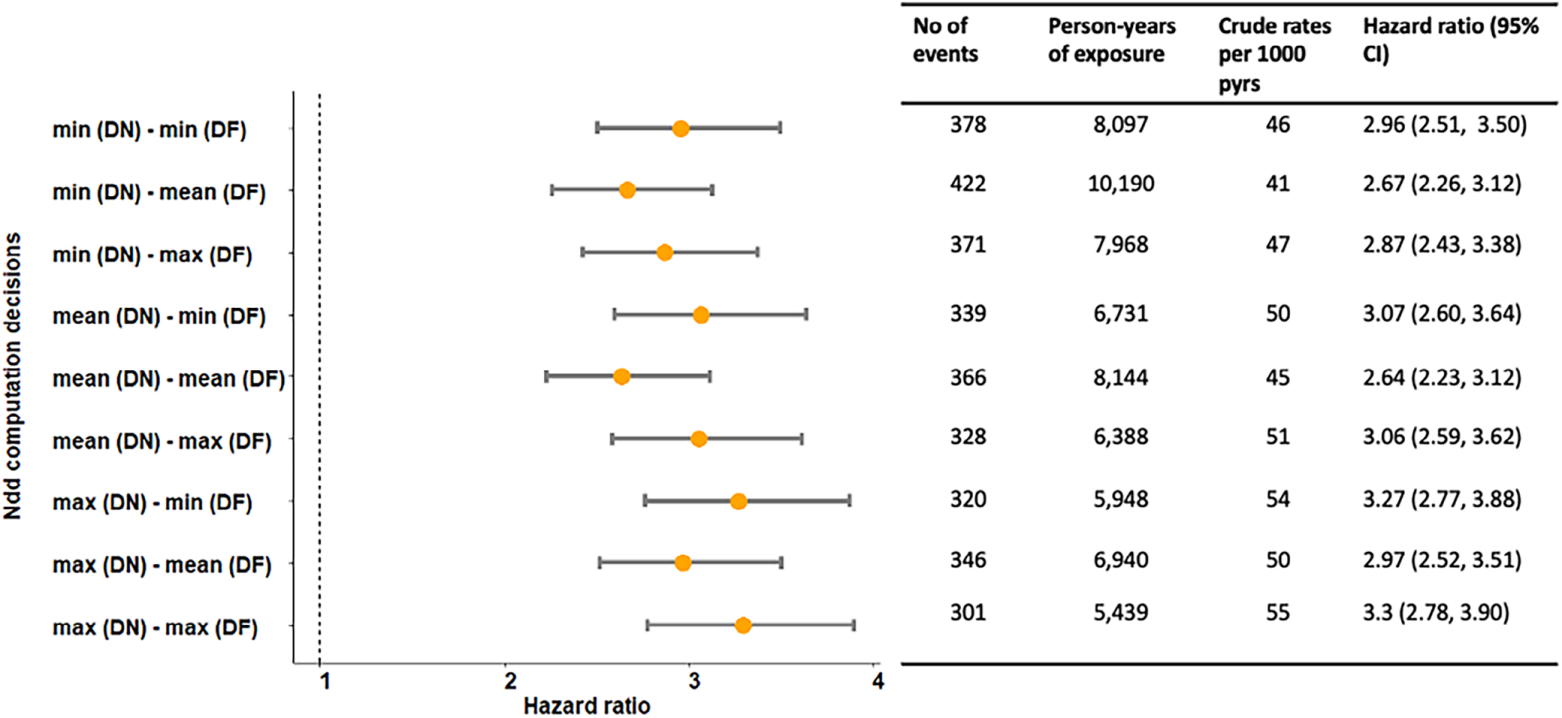
Influence of ndd computation on the association between opioid prescription and fracture events. CI, confidence intervals; DF, drug frequency; DN, drug number; HR, hazard ratio; ndd, numeric daily dose; no, number; max, maximum; min, minimum; pys, person years.

A total of 964 fracture events were observed during the whole study window. The observed number of fractures during exposed person time ranged from 303 (for maximum‐DN and maximum‐DF) to 415 (for minimum‐DN and mean‐DF) (Figure 5). The distribution of crude rates as a function of the decisions ranged from 42 to 54 per 1000 person years, while HR ranged from 2.71 (95% CI, 2.31, 3.12) to 3.24 (95% CI, 2.76, 3.82) (Figure 5). Full results of the primary analysis are presented in Table S1. Examples of how CPRD text extraction and *doseminer* extraction differ is presented in Table S2. If *doseminer* was not used at all and the rest of the drug preparation decisions remain consistent with the above, the HR was 3.09 (2.63, 3.64).

**FIGURE 6:**
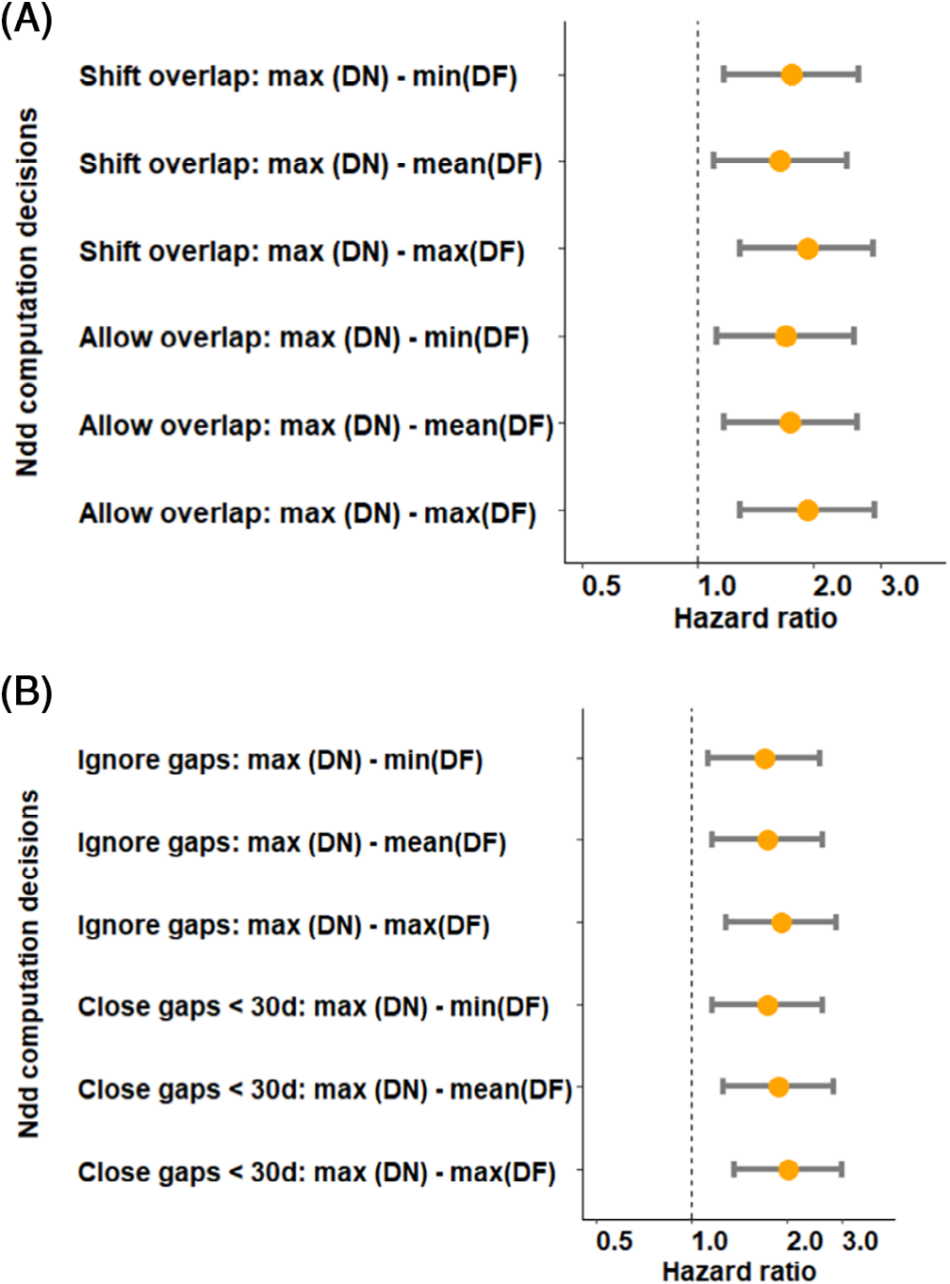
Sensitivity analysis to assess the effect of different drug preparation decisions regarding handling overlapping prescriptions and short gaps between prescriptions. (A) Altering the decisions on handling overlapping prescriptions (Decision 10) without altering the path at decision 11. (B) Altering the decision on handling gaps between prescriptions (Decision 11) while retaining the original path at decision 10. For clarity in presentation, we only considered 2 out of the 4 possible options for Decision 11 and fixed the dose number to its maximum value in Figure 5A,B. DF, drug frequency; DN, drug number; max, maximum; min, minimum.

To evaluate the effect of varying decisions regarding overlapping prescriptions and handling the gap between multiple prescriptions, we restricted the cohort to 4141 individuals with at least three opioid prescriptions (total of 31 208 prescriptions) in a sensitivity analysis. The median number of prescription among this cohort was 10 (IQR: 5–18). Figure 6 illustrates selected differences in point estimate observed when altering these decisions for overlapping prescriptions (Panel A) and short prescription gaps (Panel B), with the hazard ratios not altering to a significant degree (full results presented in Table S3).

## 5 DISCUSSION

We have developed an algorithm and associated R packages to process raw prescription data transparently from narrative instructions together with associated coded prescription data to generate prepared medication exposure data for pharmacoepidemiology research. Using an applied example of opioid use on fracture risk we have illustrated how decisions during the preparation process, including decisions using inherent variability in free text instructions, can impact event rates and risk estimates. Such decisions may be especially important where medications are prescribed ‘prn’ or as required, where exposure misclassification is more likely.

The raw prescription data provided by CPRD does not provide a “stop date” of the prescription and had missing *numdays* and missing *dose duration* for 98.3% and 99.9% respectively. Due to this high proportion of missing data, we avoided the calculation of exposure duration using simply these variables, instead defining the stop date using start date + (quantity/ndd). We observed a substantial difference in person‐years of exposure when we compared drug exposure calculated using the nine different assumptions at Step 1 (computation of ndd, see Figure 1). The person‐years (pyrs) of exposure for minimum‐DN and mean‐DF decision (9832 pyrs) was about twice the person‐year exposure for the maximum‐DN and maximum‐DF (5619 pyrs) (Figure 5).

Post marketing drug safety studies have informed regulatory decisions about retaining drugs on the market, including drugs with variability in prescribing instructions such as nonsteroidal anti‐inflammatory drugs and COX‐2 inhibitors.^24^ Transparency in data preparation allows reproducible research, as well as replication of results in a different data source.^4^, ^8^ This is particularly important as the number of drug data preparation assumptions increases with inherent variability in free text instructions. In our worked example of opioids and fracture, the results derived from different ndd assumptions generated quite different drug exposure periods, but ultimately resulted in largely similar hazard ratios. This may or may not be the case for different drug and adverse event pairs. The importance of regulatory decision making, plus the daily use of pharmacoepidemiology research informing shared informed decision making for clinicians and patients, means researchers should carefully consider and explore the implications of their assumptions. This is made accessible by the sharing of reusable code as a pair of R packages.

There are some limitations of this work. The true “gold standard” of drug exposure is only available directly from the patient, so we do not know which of the pathways are ‘correct’. Prescribing instructions for ‘as required’ medications intentionally incorporate flexibility allowing the patient to titrate dosage according to symptoms or clinical need, which may vary on a daily or weekly basis. Hence, it is unlikely that any single pathway provides the ‘right’ response. In the absence of patient‐generated data (as is typical), the outlined approach nonetheless provides a framework and opportunity to calculate risk estimates using transparent and therefore reproducible assumptions. We have chosen single assumptions for all prescriptions, but it would be possible to extend the decision framework to vary by prescriptions and select specific values rather than minimum, mean and maximum values in terms of DN, DI, and DF. As with other types of clinical free‐text, prescription records can often contain misspellings. The *doseminer* part of the algorithm was based on a published text‐mining system that currently does incorporate all possible versions of misspellings.^11^ Our algorithm was developed using UK data from CPRD and therefore would need to be modified appropriately when preparing data from other EHR databases or claims datasets. How this data source compares to other international data sources and jurisdictions is discussed elsewhere.^19^ The implications of drug preparation decisions may vary depending on the data source, the information available within the raw data (e.g., quantity, duration, etc.) and level of missingness. We would also expect the implications of the assumptions to vary according to the medication under study and the adverse event of interest. Options in the drug preparation process to handle instances of missingness and implausible quantities are currently restricted to mean, median and mode at both individual and population levels (Figure 3). Additionally, routes such as nasal sprays, ocular drops and others are not considered. However, the algorithm demonstrates the first steps of preparing drug exposure data transparently. It is open‐source and adaptable, thereby allowing future options to choose individual values if appropriate for the study.

The application of algorithms in this paper represents extension of previous work to clarify sequential steps taken to prepare drug exposure data from EHRs.^8^ It adds to a body of work aimed at increasing transparency in decisions required to refine measures of drug exposure, prior to embarking on statistical analysis.^25^, ^26^ The current algorithm does not derive drug dose, which may be especially important in studies where is a likely dose‐dependent effect on the outcome. Planned extensions of this work include calculating drug dose incorporating a range of assumptions and performing a multiverse analysis that includes testing a full‐range of variability in drug preparation decisions and their impact on eventual results. The recommendation of increasing transparency of the raw data cleaning and preparation process leading to exposed or unexposed episodes of person time, is advocated and endorsed by the RECORD‐PE representing current best practice standards.^4^ This pair of R packages represents a step toward achieving this recommendation.

In conclusion, we have illustrated how the assumptions made from the narrative text prescribing instructions can lead to variability in drug exposure estimation and prevent misclassification, through an applied example of opioids use on fracture risk. Such assumptions are especially important where medicines are administered on an as required basis or where there is variability within the prescribing directions. Increasing transparency of assumptions during the data preparation stage would allow greater reproducibility and more effective comparison of published results.

## AUTHOR CONTRIBUTIONS

Meghna Jani, Belay Birlie Yimer and William G. Dixon designed the study. R package development and analysis was led by Belay Birlie Yimer (*drugprepr*) and David Selby (*doseminer*). The first manuscript draft was written by Meghna Jani. Meghna Jani, Belay Birlie Yimer, David Selby, Mark Lunt, Goran Nenadic, and William G. Dixon helped revise the paper, interpret the findings, and approved the final version.

## FUNDING INFORMATION

The authors received no specific funding for this work. MJ is funded by a National Institute for Health and Care Research (NIHR) Advanced Fellowship (NIHR301413). The views expressed in this publication are those of the authors and not necessarily those of the NIHR or the Department of Health and Social Care. The work was supported by the Versus Arthritis Centre for Epidemiology, the authors' host institution (grant number 20380).

## CONFLICT OF INTEREST STATEMENT

William G. Dixon has received consultancy fees from Google, unrelated to this work. The rest of the authors have no conflicts to declare.

## ETHICS STATEMENT

The study was approved by the CPRD's Independent Scientific Advisory Committee (approval number: 16_278). Individual patient consent was not applicable for this study.

## Supporting information


**Data S1:** Supporting Information

## Data Availability

The data used for the case study are available through The Clinical Practice Research Datalink (CPRD) (https://www.cprd.com/, contact for data queries: enquires@cprd.com) for researchers who meet criteria for access to confidential data. All other packages, scripts and code lists are available as referenced in the manuscript on CRAN and Github.
