# Supplementary material for: “Take up to eight tablets per day”: Incorporating free‐text medication instructions into a transparent and reproducible process for preparing drug exposure data for pharmacoepidemiology
Source: Pharmacoepidemiol Drug Saf. 2023 Feb 11;32(6):651–60. doi: 10.1002/pds.5595 (PMC10947089; doi:10.1002/pds.5595)
Supplement: Supplementary file 1 — Data S1: Supporting Information [file PDS-32-651-s001.docx]

**Supplementary Appendix**

**Supplementary Figure 1: Flow diagram of the doseminer algorithm**

**Stage 1: Clean prescription**


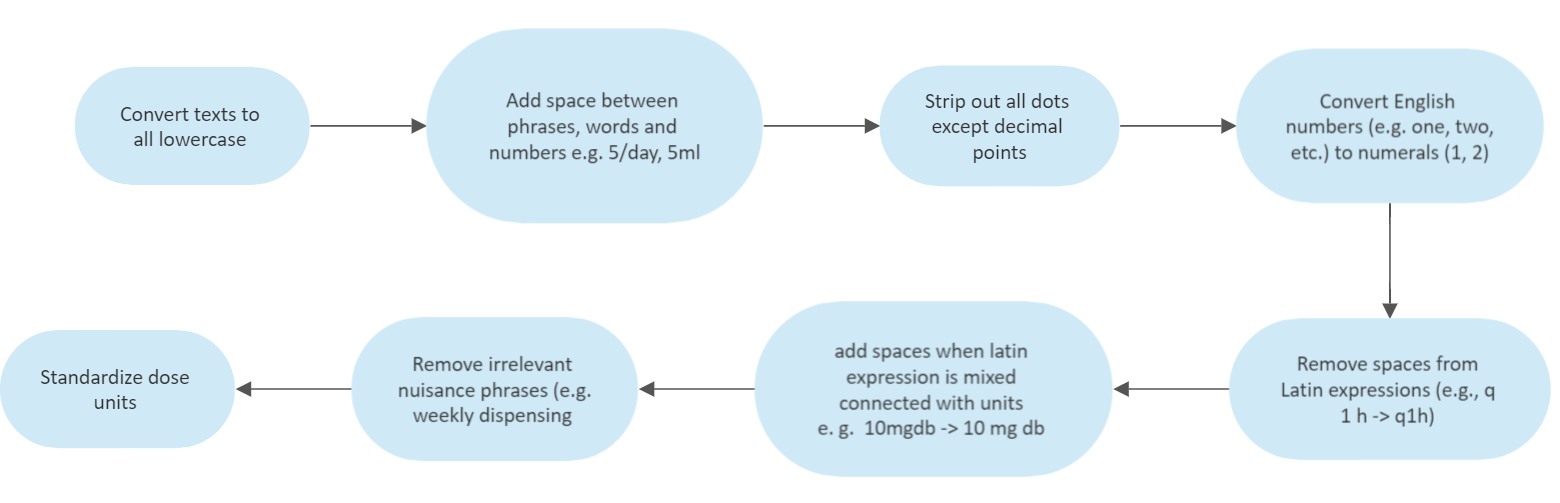


**Stage 2: Extract prescription**


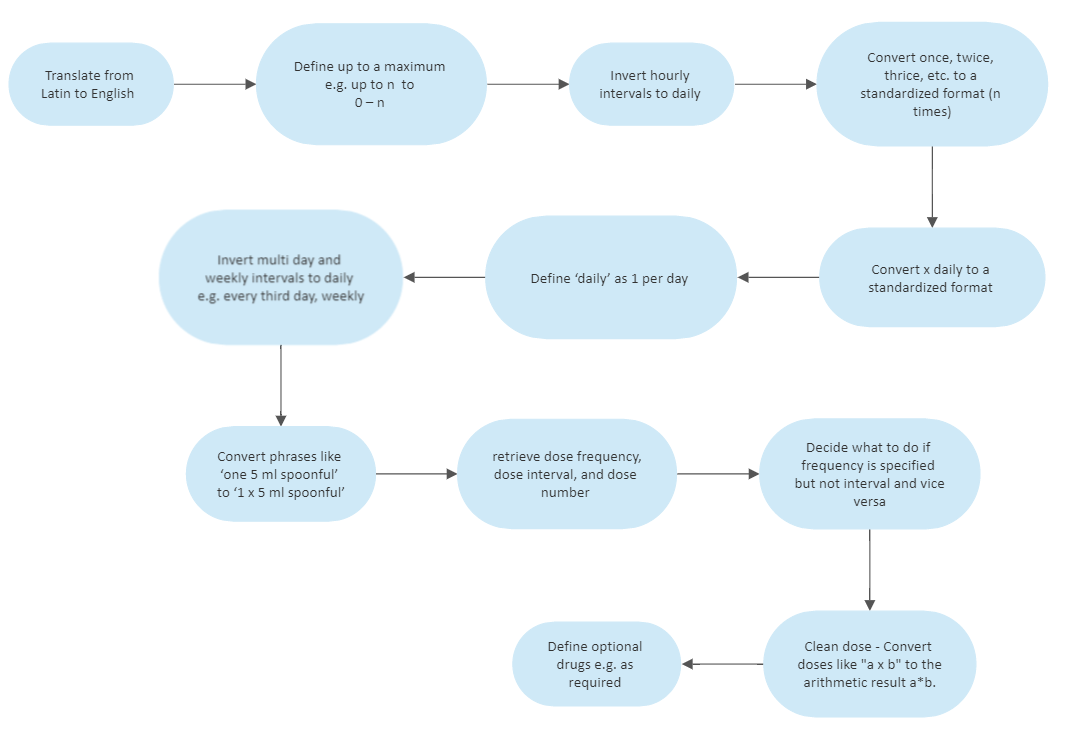


**Supplementary Figure 2: Derived exposure durations for one single prescription assuming a minimum drug frequency of 1**

**
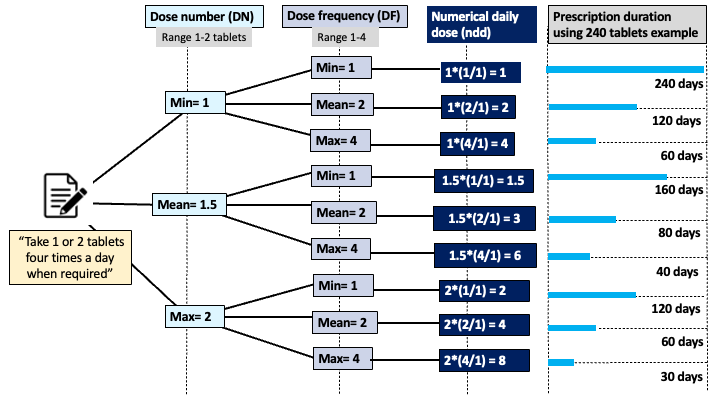
**

**Supplementary Table 1: Primary analysis results**

| Decisions | Status | Person-years | Events | Crude rates per 1000 Person- years | HR (95%CI) |
| --- | --- | --- | --- | --- | --- |
| Min (DN) - min(DF) | Exposed | 7674.65 | 366 | 47.68 | 2.97 (2.53, 3.49) |
|  | Unexposed | 56172.38 | 597 | 10.63 |  |
| Min (DN) - mean(DF) | Exposed | 9832.56 | 415 | 42.20 | 2.71 (2.31, 3.18) |
|  | Unexposed | 54104.94 | 549 | 10.15 |  |
| Min (DN) - max(DF) | Exposed | 7950.20 | 373 | 46.92 | 2.93 (2.50, 3.45) |
|  | Unexposed | 55937.59 | 591 | 10.57 |  |
| Mean (DN) - min(DF) | Exposed | 6526.54 | 329 | 50.41 | 3.01 (2.56, 3.55) |
|  | Unexposed | 57309.64 | 634 | 11.06 |  |
| Mean (DN) - mean(DF) | Exposed | 7995.37 | 366 | 45.78 | 2.74 (2.33, 3.23) |
|  | Unexposed | 55891.58 | 598 | 10.69 |  |
| Mean (DN) - max(DF) | Exposed | 6510.04 | 331 | 50.84 | 3.05 (2.59, 3.60) |
|  | Unexposed | 57365.31 | 633 | 11.03 |  |
| Max (DN) - min(DF) | Exposed | 5798.87 | 311 | 53.63 | 3.24 (2.76, 3.82) |
|  | Unexposed | 58037.43 | 652 | 11.23 |  |
| Max (DN) - mean(DF) | Exposed | 6964.00 | 342 | 49.11 | 2.91 (2.47, 3.42) |
|  | Unexposed | 56915.04 | 622 | 10.92 |  |
| Max (DN) - max(DF) | Exposed | 5619.30 | 303 | 53.92 | 3.20 (2.71, 3.77) |
|  | Unexposed | 58255.19 | 661 | 11.35 |  |

**Supplementary Table 2:**

**CPRD and doseminer daily dose comparison**

| Free text | **CPRD text extraction** | | | | **Doseminer extraction** | | |
| --- | --- | --- | --- | --- | --- | --- | --- |
|  | Daily dose | Dose number | Dose frequency | Dose interval | Dose number | Dose frequency | Dose interval |
| 1-2 capsules up to four times a day | 6 | 1.5 | 4 | 1 | 1-2 | 0-4 | 1 |
| 2 tabs 4-6HRLY up to four times a day | 4 | 0 | 1 | 0.25 | 2 | 4-6 | 1 |
| Take one or two four times a day | 6 | 1.5 | 4 | 1 | 1-2 | 4 | 1 |
| 1 or 2 four times a day when required | 6 | 1.5 | 4 | 1 | 1-2 | 4 | 1 |


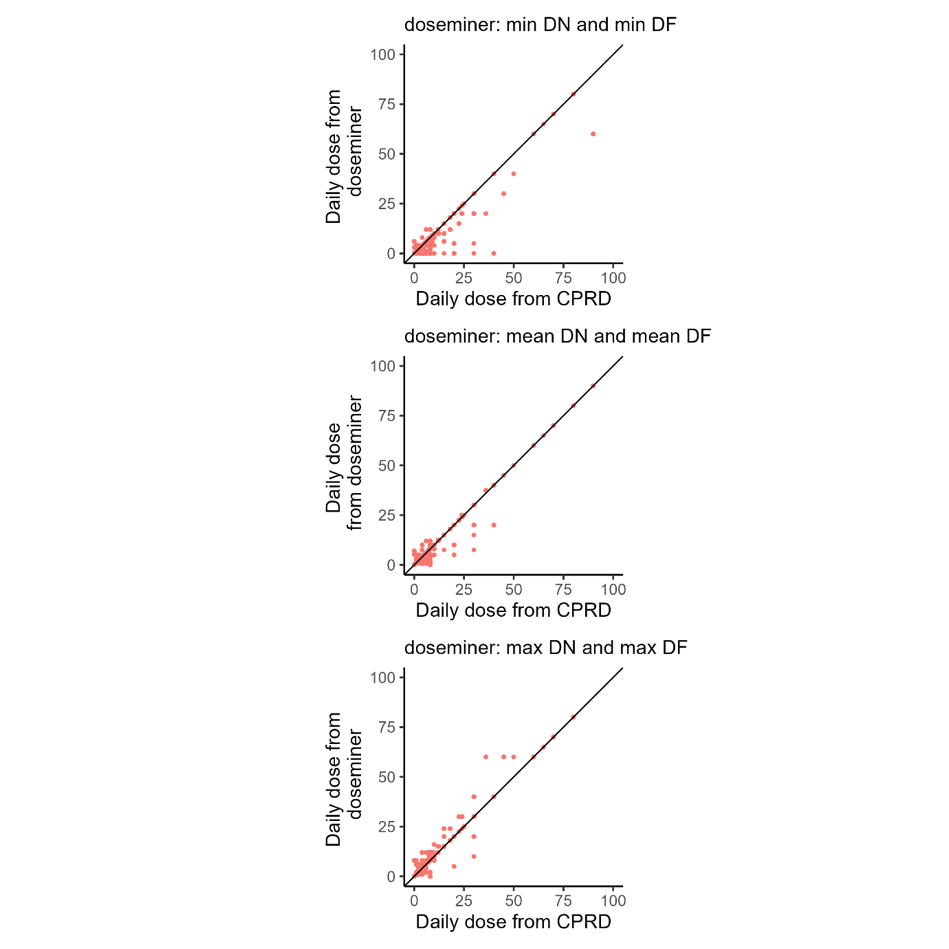


**Analysis using CPRD daily dose without doseminer**

| **HR (95% CI)** | **Events exposed (n)** | **Person years exposed** | **Events unexposed (n)** | **Person years unexposed** |
| --- | --- | --- | --- | --- |
| 3.09 (2.63, 3.64) | 332 | 6,486 | 632 | 57,386 |

Other drug preparation decisions set the same as the main analysis

**Supplementary Table 3:**

**Sensitivity analysis results: Impact of decision on overlapping prescriptions and small gaps**

| Decisions | Number of events during the unexposed period | Number of events during an exposure period | Person-years of unexposed period | Person-years of exposure | HR (95% CI) |
| --- | --- | --- | --- | --- | --- |
| Impact of decisions on overlapping prescriptions | | | | | |
| Allow overlap: max (DN) - min(DF) | 71 | 56 | 3421.795 | 959.2348 | 1.70 (1.13, 2.55) |
| Allow overlap: max (DN) - mean(DF) | 65 | 63 | 3273.769 | 1119.325 | 1.74 (1.16, 2.59) |
| Allow overlap: max (DN) - max(DF) | 72 | 56 | 3500.088 | 891.8919 | 1.93 (1.29, 2.88) |
| Shift overlap: max (DN) - min(DF) | 69 | 58 | 3394.45 | 1028.231 | 1.75 (1.67, 2.62) |
| Shift overlap: max (DN) - mean(DF) | 65 | 63 | 3229.665 | 1210.255 | 1.64 (1.10, 2.44) |
| Shift overlap: max (DN) - max(DF) | 71 | 57 | 3480.281 | 948.2875 | 1.93 (1.29, 2.87) |
| Impacts of decisions on small gaps | | | | | |
| Close gaps < 30d: max (DN) - min(DF) | 60 | 67 | 3141.02 | 1252.142 | 1.74 (1.16, 2.59) |
| Close gaps < 30d: max (DN) - mean(DF) | 55 | 73 | 3040.007 | 1363.837 | 1.88 (1.26, 2.80) |
| Close gaps < 30d: max (DN) - max(DF) | 59 | 69 | 3198.94 | 1205.644 | 2.01 (1.36, 2.99) |
| Ignore gaps: max (DN) - min(DF) | 71 | 56 | 3421.795 | 959.2348 | 1.69 (1.13, 2.55) |
| Ignore gaps: max (DN) - mean(DF) | 65 | 63 | 3273.769 | 1119.325 | 1.74 (1.67, 2.60) |
| Ignore gaps: max (DN) - max(DF) | 72 | 56 | 3500.088 | 891.8919 | 1.92 (1.28, 2.87) |

In the case of overlapping prescriptions, the extracted information from the free text will be retained from both prescriptions. In this paper, we are only dealing with on/off drug exposure. If the interest is in dose strength (e.g., 30mg), then one would have to consider the differences between the two prescriptions (if any).
